# Combining immunoscore and tumor budding in colon cancer: an insightful prognostication based on the tumor-host interface

**DOI:** 10.1186/s12967-024-05818-z

**Published:** 2024-12-02

**Authors:** T. S. Haddad, J. M. Bokhorst, M. D. Berger, L. v. d. Dobbelsteen, F. Simmer, F. Ciompi, J. Galon, J. v. d. Laak, F. Pagès, I. Zlobec, A. Lugli, I. D. Nagtegaal

**Affiliations:** 1 Radboud University Medical Center, Nijmegen, Netherlands; 2 Department of Medical Oncology, Inselspital, Bern University Hospital, University of Bern, Bern, Switzerland; 3 Centre de Recherche Des Cordeliers, Sorbonne Université, Université Paris Cité, 75006 Paris, France; 4 Institute of Tissue Medicine and Pathology, University of Bern, Bern, Switzerland; 5 Department of Pathology, RadboudUMC, 6525 GA Nijmegen, The Netherlands

**Keywords:** Immunoscore, Tumor budding, Colon cancer, AI

## Abstract

**Background:**

Tumor Budding (TB) and Immunoscore are independent prognostic markers in colon cancer (CC). Given their respective representation of tumor aggressiveness and immune response, we examined their combination in association with patient disease-free survival (DFS) in pTNM stage I-III CC.

**Methods:**

In a series of pTNM stage I-III CCs (n = 654), the Immunoscore was computed and TB detected automatically using a deep learning network. Two-tiered systems for both biomarkers were used with cut-offs of 25% and ten buds for Immunoscore and TB according to clinical guidelines, respectively. Associations of Immunoscore with TB with 5-year DFS were examined using Kaplan–Meier survival analysis in addition to multivariable modeling and relative contribution analysis using Cox regression.

**Results:**

Immunoscore and TB independently are prognostic with hazard ratio (HR) = 2.0, 95% confidence interval (CI) 1.4–2.8 and HR 2.5, with 95% CI 1.4–4.5, respectively; *P* value < 0.0001. By combining Immunoscore with TB, patients with Immunoscore Low, TB High tumors had a significantly poorer DFS (HR 5.6, 95% CI 2.6–12.0; *P* value < 0.0001) than those with Immunoscore High, TB Low tumors. The combined Immunoscore with TB score was independently prognostic (*P* value = 0.009) in comparison to N-stage, T-stage, and MSI. Immunoscore with TB had the highest relative contribution (35%) to DFS in pTNM stage I-II CCs.

**Conclusions:**

The association of Immunoscore and TB with patient survival suggests that both biomarkers are complementary and should be interpreted in combination to identify high-risk Stage I-II patients who should be considered for adjuvant therapy or further diagnostic testing.

**Supplementary Information:**

The online version contains supplementary material available at 10.1186/s12967-024-05818-z.

## Introduction

The field of immunology has grown rapidly and shown to play an important role in the pathology and treatment of solid tumors. Immune infiltrate has been extensively studied and is considered to be a strong prognostic factor in colon cancer (CC) [1–8]. The rise of digital pathology has ushered in a new era of computational techniques where the immune assessment can now be translated into clinical practice. Franck Pages, Jérôme Galon and colleagues were the first to develop a standardized, immune-based assay formally known as Immunoscore^®^ (Veracyte) [9]. Immunoscore utilizes densities of CD3 + T-cells and CD8 + cytotoxic T-cells within the tumor and invasive margin. Patients are stratified into tiered categories which ranks their risk of recurrence and helps guide clinical decision-making and monitoring. Having been validated in multiple large international consortium studies, Immunoscore is highly effective at predicting risk of recurrence, response to chemotherapy and neoadjuvant treatment in CC [9–13].

Another strong prognostic marker in solid tumors is tumor budding (TB). Standardized by the International Tumor Budding Consensus Conference (ITBCC), TB is defined as single cells and isolated clusters of up to four cells at the tumor invasive front [14, 15]. TB is an independent predictor of lymph node metastasis (LNM) and cancer-related deaths in CC patients [16]. TB’s prognostic value has been validated in multiple large multi-center clinical trials [17, 18]. With artificial intelligence (AI) being developed for clinical applications, digital pathology is bringing emerging technologies such as AI ever closer to clinical practice. The International Tumor Budding consortium (IBC) has developed an effective AI model for automated detection of TB in H&E whole-slide images [19]. Analogous to ITBCC guidelines, this model is efficient, reproducible and has been validated in large multi-centric cohorts.

Both Immune response and TB have been incorporated into the World Health Organization (WHO) Classification of Digestive System Tumours (2019) [20] as essential complements to the traditional pTNM staging system, yet their prognostic value in combination has yet to be explored.

Based on the “attacker-defender” model [21] which better reflects the interaction between a tumor-related and host-related biomarker, our aim was to combine TB and immune infiltration in a large cohort of pTNM stage I-III CC patients, thereby attempting to create the first fully digitized clinical representation of the tumor-host interaction. Considering the significant clinical implications of Immunoscore and TB in CC diagnostics, we determine whether their combination will yield greater prognostic relevance than independently as well as provide insight into the underlying biology of CC.

## Methods

### Study population

A cohort of primary tumors from patients with pTNM stage I-III CC who did not receive neoadjuvant treatment was established by the Immunoscore International consortium [9]. A subset of cases from this cohort based on tissue and data availability (491 patients from the Netherlands, 163 patients from Switzerland) was used for this study. Disease-Free Survival (DFS) was calculated from initial treatment to diagnosis of first relapse with 5-year follow-up time. This study was approved by ethical review boards at each center (approval numbers 2017-3603, 2017-01803).

### Immunoscore assessment

Immunoscore was assessed as previously published [9]. Densities of CD3 + and CD8 + T cells within the tumor core and invasive margin were determined using a previously developed Immunoscore module. For each case, CD3 + and CD8 + cell densities within the tumor core and invasive margin were converted into percentiles. The mean of the four percentiles (CD3CT, CD3IM, CD8CT, CD3IM) was calculated and converted into an Immunoscore. A mean-percentile of 25% was set as a cut-off to distinguish between Immunoscore Low and High in accordance with the two-tiered Immunoscore system as previously defined [9].

### Tumor budding assessment

**Fig. 1:**
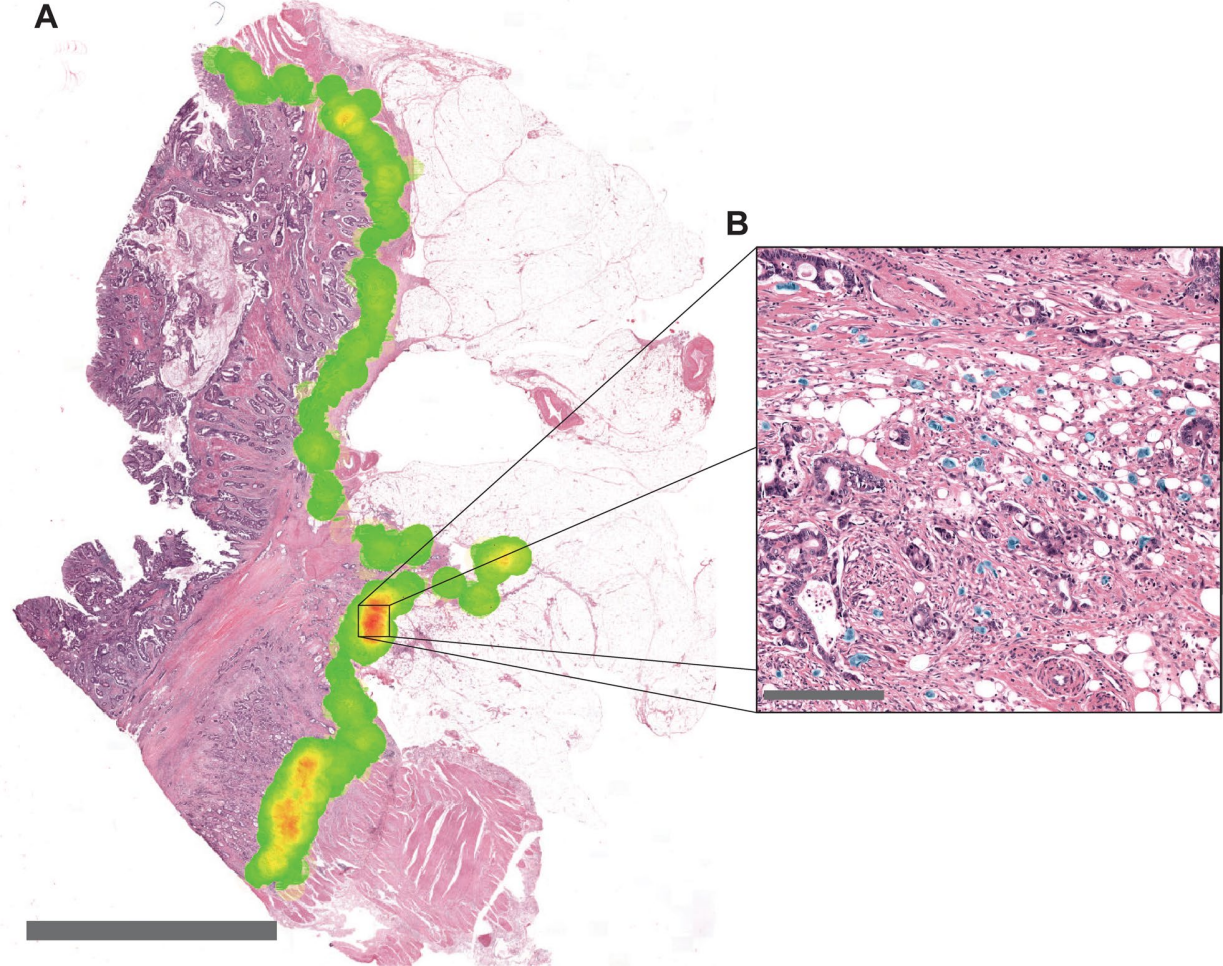
Tumor Budding (TB) assessment using automated detection of TB in HE using deep Learning. (**A**) An H&E slide processed with the automated tumor budding detection algorithm in HE. The heatmap across the invasive front represents hotspot detection where the densities of tumor buds along the invasive margin are determined. (**B**) Detection of TB within the hotspot with an area of 0.785 mm^2^. Automatically detected TB is denoted with the blue overlay

TB was assessed using a deep learning network for automated detection of TB in HE whole-slide images [19]. For each case, TB was scored automatically analogous to ITBCC guidelines [14]. An invasive margin of 1000 μm was determined using an invasive margin detection algorithm. TB was scored across the entire invasive margin and a hotspot of 0.785 mm^2^ was determined using a hotspot detection algorithm (Fig. 1A). The number of TB within the hotspot was determined to produce a TB score (Fig. 1B). A cut-off of ten buds for high-grade TB was used to distinguish between low and high-grade TB in accordance with ITBCC guidelines for routine diagnostic assessment of TB.

### Statistical analysis

Statistical analyses were performed using R v4.2.0. Associations between Immunoscore, TB and time-to-event outcomes were evaluated using the Cox proportional hazards model. Multivariable Cox models were used to assess DFS by Immunoscore with TB having adjusted for covariates (sex, T/N-stage, tumor site, lymphatic invasion and perineural invasion). Hazard ratios (HR) and 95% confidence intervals (CI) are reported. *P* values are reported and considered statistically significant when *P* < 0.05. Demographics and disease characteristics were compared by χ^2^ and Kruskal–Wallis when appropriate. Relative contributions of each variable to DFS were calculated using χ^2^ from Harrel’s ‘rms’ package in R (https://hbiostat.org/R/rms/).

## Results

### Patient characteristics

Within our study cohort (n = 654), the mean patient age was 68 (SD = 13) with a median follow-up of 48 months. The median number of lymph nodes dissected was 12. Patients were stratified into one of four groupings depending on their combined scores (Immunoscore Low, TB High; Immunoscore Low, TB Low; Immunoscore High, TB High; Immunoscore High, TB Low). Patient characteristics based on these stratifications are shown in Table 1. A majority of patient tumors were categorized as Immunoscore High, TB High (66%) with a minority as Immunoscore Low, TB Low (4%). Patient tumors categorized as Immunoscore High, TB Low or Immunoscore Low, TB High at similar percentages (14% and 16.5%, respectively). Fairly similar distributions can be seen across all groups with regards to age, sex, and tumor site. The highest prevalence of mucinous tumors (46.2%) was observed in the smallest group of patients (Immunoscore Low, TB Low, 4%) and the lowest prevalence of mucinous tumors (16.9%) within the largest group (Immunoscore High, TB High, 66%) (*P* = 0.0005). A significantly higher percentage of T3/T4 tumors were considered to be Immunoscore Low, TB High (93.5%) compared with Immunoscore High, TB Low (55.7%) (*P* < 0.0001). Similarly significant trends can be seen with N-stage as percentages of nodal positivity increases across all four groups from Immunoscore High, TB Low (13.8%) to Immunoscore Low, TB High (54.6%) (*P* < 0.001). Twenty-five percent of Immunoscore High, TB Low tumors were considered MMR deficient in comparison to only 12% of Immunoscore Low, TB high tumors (*P* = 0.049). Lymphatic invasion was observed in 55.6% of patients with Immunoscore Low, TB High tumors when compared to only 11.4% of patients with Immunoscore High, TB Low tumors (*P* < 0.0001).

**Table 1:** Clinicopathological features of patients stratified by Immunoscore with Tumor Budding (TB)

	Total		Immunoscore High, TB Low		Immunoscore Low, TB Low		Immunoscore High, TB High		Immunoscore Low, TB High		P value
Variables	(n = 654)		(n = 88)		(n = 26)		(n = 432)		(n = 108)		
Age (years), n (%)											0.68^c^
≤ 70	324	(49.5)	44	(50)	14	(53.8)	218	(50.5)	48	(44.4)	
> 70	330	(50.5)	44	(50)	12	(46.2)	214	(49.5)	60	(55.6)	
Sex, n (%)											0.74^c^
Male	337	(51.5)	43	(48.9)	12	(46.2)	222	(53.4)	60	(55.6)	
Female	317	(48.5)	45	(51.1)	14	(53.8)	210	(48.6)	48	(44.4)	
Tumor site, n (%)											0.38^c^
Proximal	352	(53.8)	46	(52.3)	16	(61.5)	237	(56.6)	53	(49.1)	
Distal	302	(46.2)	42	(47.7)	10	(38.5)	195	(43.4)	55	(50.9)	
Tumor type, n (%)											0.0005^c^
Adenocarcinoma	518	(79.2)	64	(72.7)	14	(53.8)	359	(83.1)	81	(75)	
Mucinous	136	(20.8)	24	(27.3)	12	(46.2)	73	(16.9)	27	(25)	
T stage, n (%)											< 0.0001^c^
T1 + T2	122	(18.7)	39	(44.3)	4	(15.4)	72	(16.7)	7	(6.5)	
T3 + T4	532	(81.3)	49	(55.7)	22	(84.6)	360	(83.3)	101	(93.5)	
N stage, n (%)											< 0.0001^c^
N0	378	(57.8)	75	(85.2)	18	(69.2)	236	(54.6)	49	(45.4)	
N +	276	(42.2)	13	(14.8)	8	(30.8)	196	(45.4)	59	(54.6)	
pTNM stage, n (%)											< 0.0001^k^
I	102	(15.6)	36	(40.9)	4	(15.4)	57	(13.2)	5	(4.7)	
II	276	(42.2)	39	(44.3)	14	(53.8)	179	(41.4)	44	(40.7)	
III	276	(42.2)	13	(14.8)	8	(30.8)	196	(45.4)	59	(54.6)	
MSI status, n (%)											0.049^c^
Proficient MMR	482	(73.7)	62	(70.4)	20	(77)	312	(72.2)	88	(81.5)	
Deficient MMR	147	(22.5)	22	(25)	5	(19.2)	107	(24.8)	13	(12)	
Unknown	25	(3.8)	4	(5.6)	1	(3.8)	13	(3)	7	(6.5)	
Differentiation, n (%)											0.02^c^
Low grade	495	(75.7)	77	(87.5)	22	(84.6)	319	(73.8)	77	(71.3)	
High grade	159	(24.3)	11	(12.5)	4	(15.4)	113	(26.2)	31	(28.7)	
Lymphatic Invasion, n (%)											< 0.0001^c^
No	451	(65.1)	78	(88.6)	18	(69.2)	307	(71.1)	48	(44.4)	
Yes	203	(34.9)	10	(11.4)	8	(30.8)	125	(28.9)	60	(55.6)	
Perineural Invasion, n (%)											0.001^c^
No	587	(89.8)	85	(96.6)	23	(88.5)	392	(90.7)	87	(80.6)	
Yes	67	(10.2)	3	(3.4)	3	(11.5)	40	(9.3)	21	(19.4)	

P-values in bold are statistically significant

TB, Tumor Budding; MSI, Microsatellite Instability; MMR, Mismatch Repair

^C^Chi-squared test

^k^Kruskal Wallis test

### Associations of immunoscore and tumor budding with disease-free survival

**Fig. 2:**
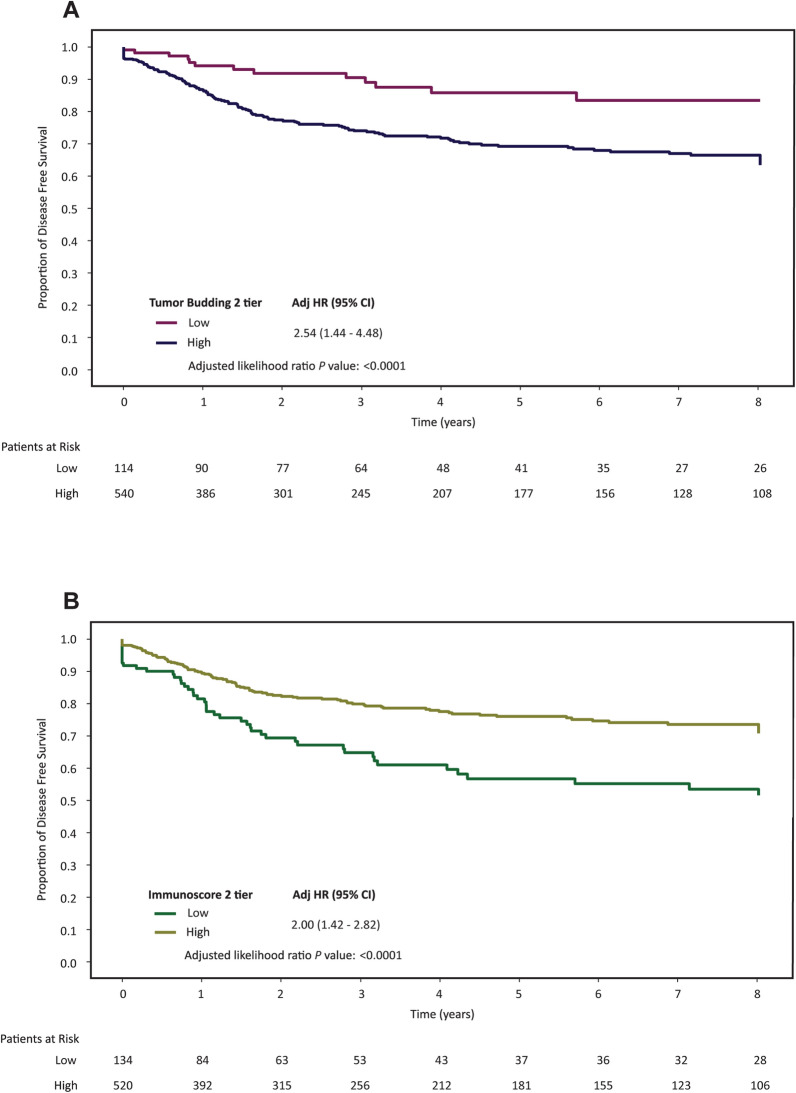
Association of Immunoscore and Tumor Budding (TB) with DFS for patients with Stage I-III colon cancer based on Univariable Analysis. (**A**) TB and (**B**) Immunoscore in overall cohort. HR, hazard ratio; CI, confidence interval

The prognostic values of Immunoscore and TB independently were evaluated (Fig. 2A, B). Regarding Immunoscore, 20.5% of patients presented with Immunoscore High and 79.5% presented with Immunoscore Low. Similarly, 17.4% of patient tumors were considered TB Low and 82.6% TB High. When comparing DFS rate, patients whose tumors were Immunoscore Low [HR 2.0 (95% CI 1.4–2.8); *P* < 0.0001] or TB High [HR 2.5 (95% CI 1.4–4.5); *P* < 0.0001] had significantly poorer DFS than Immunoscore High or TB Low, respectively.

**Fig. 3:**
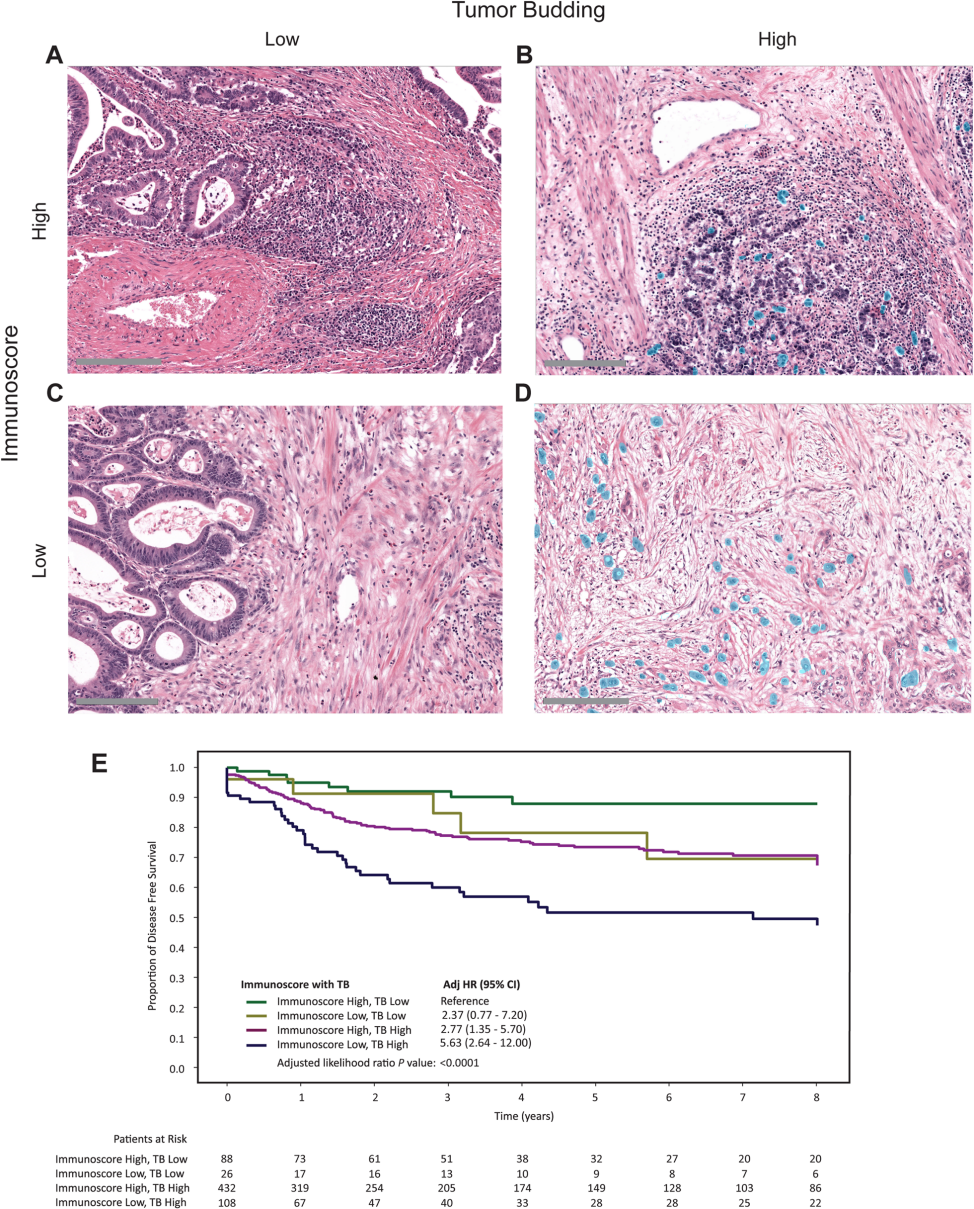
Tumor Budding (TB) and lymphocytic infiltration (as represented by Immunoscore) at the invasive front as well as Kaplan–Meier estimates of DFS for patients within the 4 Immunoscore with TB groupings. **A**-**D**. Examples of the different categories on H&E slides (**A**) Immunoscore High, TB Low; (**B**) Immunoscore Low, TB High; (**C**) Immunoscore Low, TB Low; (**D**) Immunoscore Low, TB High. Automatically detected TB is denoted with the blue overlay. (E) Kaplan–Meier estimates for DFS patients with tumors in the 4 different Immunoscore with TB groupings. HR, hazard ratio; CI, confidence interval

Using Immunoscore and TB, we created four different groups, of which examples are given in Fig. 3A–D. The prognostic value of Immunoscore with TB across the entire cohort was evaluated (Fig. 3E). Patients whose tumors were Immunoscore Low, TB High had the poorest DFS (HR 5.6, 95% CI 2.6–12.0; *P* value < 0.0001) in reference to patients whose tumors were Immunoscore High, TB Low. Interestingly, patients whose tumors were Immunoscore Low, TB Low or Immunoscore High, TB High had similar DFS rates (HR 2.4, 95% CI 0.8–7.2 and HR 2.8, 95% 21.4–5.7, respectively; *P* value < 0.0001). For subsequent analyses, the Immunoscore Low, TB Low and Immunoscore High, TB High groups were combined to form a single Intermediate group (Supplementary Fig. 1). We repeated the analyses with alternative cut-off values with similar results (supplemental Figs. 2 and 3).

**Fig. 4:**
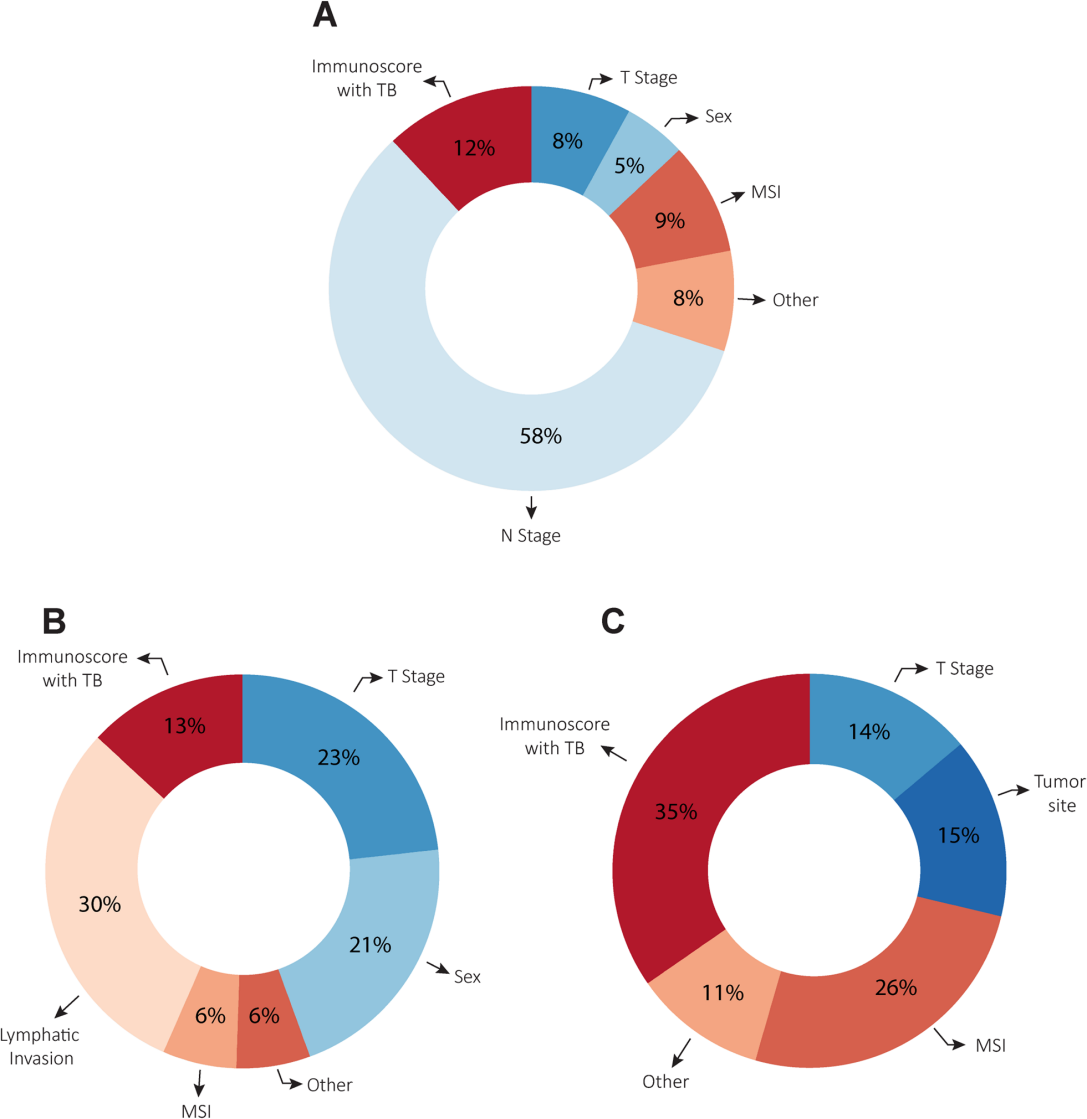
Relative contributions (%) of variables in multivariable Cox analysis for prediction of DFS in (**A**) overall cohort; and patients categorized into (**B**) pTNM stage III and (**C**) pTNM stage I-II groups. ‘Other’ includes age, sex, tumor type, differentiation, perineural invasion, lymphatic invasion, and tumor site which had relative contributions less than 5%. DFS, disease-free survival

A forest plot was created based on results of the multivariate regression analysis of Immunoscore with TB and tumor-related variables that were significantly associated with DFS in the patient population based on univariable analysis (Supplementary Tables 1, 2). Within the cohort, Immunoscore with TB as well as N-stage, T-stage and MSI were each significantly and independently associated with patient DFS. Upon analysis of the relative contribution of each variable to DFS based on multivariate analysis within the entire cohort, Immunoscore with TB was second only to N-stage in association to DFS (Fig. 4A). In pTNM stage III tumors, the top contributors were lymphatic invasion (30%), T-stage (23%), sex (21%) and Immunoscore with TB (13%) (Fig. 4B). Among pTNM stage I-II tumors, Immunoscore with TB had the highest relative contribution to DFS (35%) in comparison to MSI (26%) and all other variables (Fig. 4C). The exact HR values are given in supplemental Tables 3–5. In supplemental Fig. 4 we show consistency for all three categories over nodal stage.

## Discussion

In a large cohort of pTNM stage I-III CC patients, the combination of TB quantified digitally and inflammation represented by Immunoscore is more prognostic than either factor independently when comparing HR from univariable analysis (Immunoscore [HR 2.0 (95% CI 1.4–2.8)]; TB [HR 2.5 (95% CI 1.4–4.5)]; Immunoscore with TB [HR 5.6, 95% CI 2.6–12.0)];* P* < 0.0001). Immunoscore and TB are complementary and consider tumor and host-related factors to accurately reflect the biology of both the tumor and tumor microenvironment. Automated detection techniques resolve the issue of interobserver variability that comes with manual biomarker assessment [22] and clearly reflect the attacker-defender model. First described in Lugli et al. [21], the attacker-defender model shows that the ratio of invading TBs and defending lymphocytes is more prognostic than either feature alone. Several recent studies have investigated TB while incorporating lymphocytic infiltration and provided strong evidence for the attacker-defender model [23–28]. Through the current study, we were able to accurately reflect the attacker-defender model using a fully digital approach and clinically validated biomarkers which are significantly associated with DFS [9, 10, 12, 17, 18, 29]. This allows implementation in clinical practice.

Immunoscore with TB can effectively stratify patients, with patients whose tumors are Immunoscore Low, TB High having a markedly shorter five-year DFS (52% versus 88%). Patients with a high Immunoscore, even with high TB, had a significantly better outcome compared to patients having tumors with high TB and a low Immunoscore. The fact that patients with Immunoscore Low, TB Low and Immunoscore High, TB High tumors have similar DFS rates deserves comment. Based on these findings, when tumor aggressiveness and immune infiltration reflected by Immunoscore with TB are either increased or decreased in the same instance, neither the tumor nor host can overcome one another. Once either the tumor or host’s immune response become predominant as reflected by Immunoscore Low, TB High or Immunoscore High, TB Low tumors, we see a significant effect on DFS, respectively.

We examined the contribution of the combination of Immunoscore with TB to patient DFS. Analysis by pTNM stage revealed that Immunoscore with TB had the highest relative contribution to DFS among stage I-II cases, surpassing MSI. With stage I and II patients typically being considered low-risk patients, our data supports the usefulness of Immunoscore with TB in determining high-risk early-stage patients. Patients with Immunoscore Low, TB High tumors are associated with tumor progression and poorer DFS (higher risk of relapse). With the inclusion of Immune response and TB in the WHO Classification of Digestive System Tumours [20], this study highlights their importance in addition to traditional histological parameters. Both biomarkers have been validated in large series [9, 12, 18]. Immunoscore is an effective prognostic indicator and predictor of response to therapy and risk of relapse [9, 10, 13, 30]. Strong evidence has established high-grade TB as an independent prognostic marker of LNM in pT1 CC [31–34], as well as recurrence and mortality in Stage II CC [35–38].

Based on our findings, Immunoscore with TB is a strong prognostic indicator that could be used to identify high-risk Stage I-II patients who should be considered for adjuvant therapy. The prognostic information provided through Immunoscore with TB can be done with tissue from surgical pathology and could be complementary to ctDNA tests which require additional sampling [39]. Conducting a clinical trial on Immunoscore with TB would be valuable.

The strengths of the study are that it includes a large cohort of pTNM stage I-III patients from a well-characterized international cohort with long-term patient outcome data. This is the first study to combine automated TB detection using deep learning with Immunoscore in a large series of CC patients. However, this is a retrospective study that requires prospective validation in an independent cohort. We use a deep learning algorithm which has not yet been implemented in current diagnostic practice. Implementing this algorithm in routine diagnostics has the potential to make TB scoring more efficient and reproducible. Veracyte has paused its efforts to commercialize Immunoscore at the moment yet continues to support its use in an academic setting.

In conclusion, patients with Immunoscore Low, TB High tumors had the poorest DFS in the overall cohort. These findings suggest that Immunoscore and TB are complementary and should be interpreted for prognostication and clinical decision making in CC.

## Supplementary Information

Supplementary material 1: Supplementary Table 1 - Forest plot based on results of univariable analysis of variables associated with DFS in overall population. Supplementary Table 2 – Forest plot based on results of multivariate analysis of variables associated with DFS in overall cohort. Variables significant in supplementary table 1 are included in this model. Supplementary Table 3 - Forest plots associated with figure 4A. All variables were included in this multivariate Cox regression model. Supplementary Table 4 - Forest plots associated with figure 4B. All variables were included in this multivariate Cox regression model. Supplementary Table 5 - Forest plots associated with figure 4C. All variables were included in this multivariate Cox regression model. Supplementary Figure 1 - Kaplan-Meier estimates of disease-free survival for patients with colon cancer with Immunoscore Low, TB High; Intermediate; Immunoscore High, TB Low. Supplementary Figure 2– Analysis of the impact of TBand Immunoscoreon patient outcome using different cut-offs. In the main manuscript the international standards for Immunoscore and TB are used. Here a median cut-off and a cut-off based on lowest Akaike information criterionare also used in univariate Cox regression analysis. Supplementary Figure 3 – Kaplan-Meier estimates of disease-free survival for patients with colon cancer grouped into four groups based on median of TB and Immunoscore scores. The p-value obtained by the log rank test is included in the plot. Supplementary Figure 4- Kaplan-Meier estimates of disease-free survival for patients with colon cancer for the different categories to evaluate consistency for nodal stage. A. Overall cohort, B. Immunoscore high, TB low, C. Intermediate, D, Immunoscore low, TB high. The p-value obtained by the log rank test is included in the plot

## Data Availability

Data will be made available upon reasonable request.
