# Supplementary material for: Combining immunoscore and tumor budding in colon cancer: an insightful prognostication based on the tumor-host interface
Source: J Transl Med. 2024 Dec 2;22:1090. doi: 10.1186/s12967-024-05818-z (PMC11610196; doi:10.1186/s12967-024-05818-z)
Supplement: Supplementary file 1 — Supplementary material 1: Supplementary Table 1 - Forest plot based on results of univariable analysis of variables associated with DFS in overall population. Supplementary Table 2 – Forest plot based on results of multivariate analysis of variables associated with DFS in overall cohort. Variables significant in supplementary table 1 are included in this model. Supplementary Table 3 - Forest plots associated with figure 4A. All variables were included in this multivariate Cox regression model. Supplementary Table 4 - Forest plots associated with figure 4B. All variables were included in this multivariate Cox regression model. Supplementary Table 5 - Forest plots associated with figure 4C. All variables were included in this multivariate Cox regression model. Supplementary Figure 1 - Kaplan-Meier estimates of disease-free survival for patients with colon cancer with Immunoscore Low, TB High; Intermediate; Immunoscore High, TB Low. Supplementary Figure 2– Analysis of the impact of TBand Immunoscoreon patient outcome using different cut-offs. In the main manuscript the international standards for Immunoscore and TB are used. Here a median cut-off and a cut-off based on lowest Akaike information criterionare also used in univariate Cox regression analysis. Supplementary Figure 3 – Kaplan-Meier estimates of disease-free survival for patients with colon cancer grouped into four groups based on median of TB and Immunoscore scores. The p-value obtained by the log rank test is included in the plot. Supplementary Figure 4- Kaplan-Meier estimates of disease-free survival for patients with colon cancer for the different categories to evaluate consistency for nodal stage. A. Overall cohort, B. Immunoscore high, TB low, C. Intermediate, D, Immunoscore low, TB high. The p-value obtained by the log rank test is included in the plot [file 12967_2024_5818_MOESM1_ESM.docx]

Supplemental figures and tables for:

**Combining Immunoscore and Tumor Budding in Colon Cancer: An Insightful Prognostication Based on the Tumor-Host Interface**

T. S. Haddad^1^, J. M. Bokhorst^1^, M. D. Berger^2^, L. v. d. Dobbelsteen^1^, F. Simmer^1^, F. Ciompi^1^, J. Galon^3^, J. v. d. Laak^1^, F. Pagès^3^, I. Zlobec^2^, A. Lugli^4*^, I. D. Nagtegaal^1*^


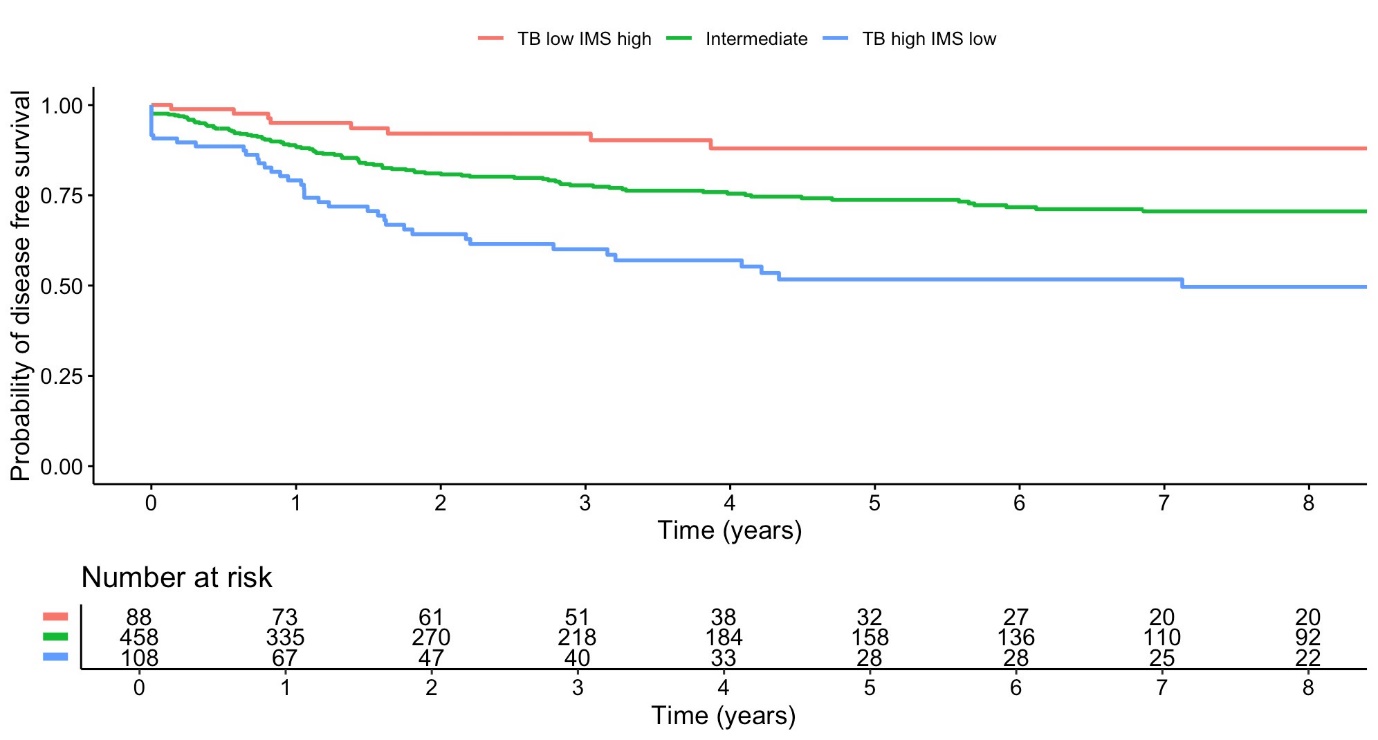


**Supplementary Figure 1 - Kaplan-Meier estimates of disease-free survival for patients with colon cancer grouped into Immunoscore Low, TB High; Intermediate (combination of Immunoscore Low, TB Low and Immunoscore High, TB High); Immunoscore High, TB Low.**


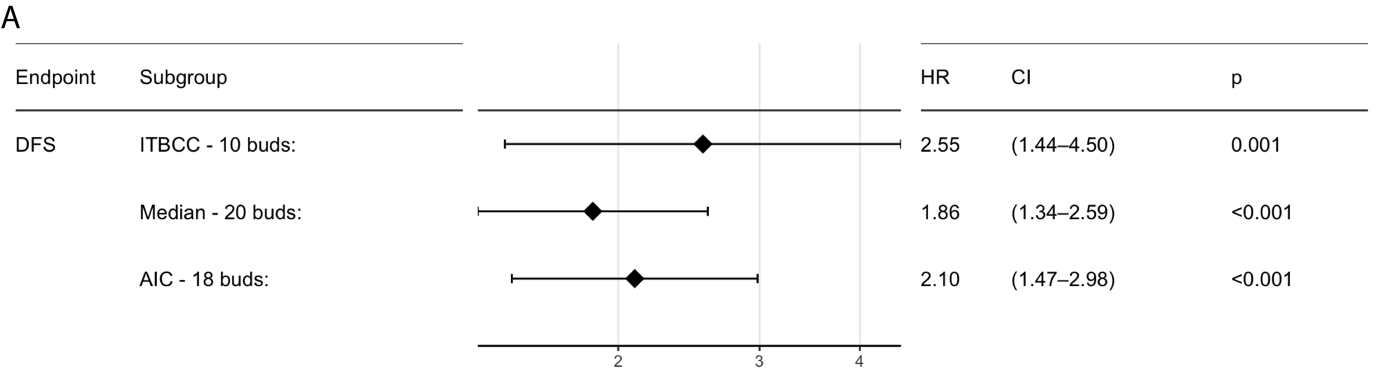


**
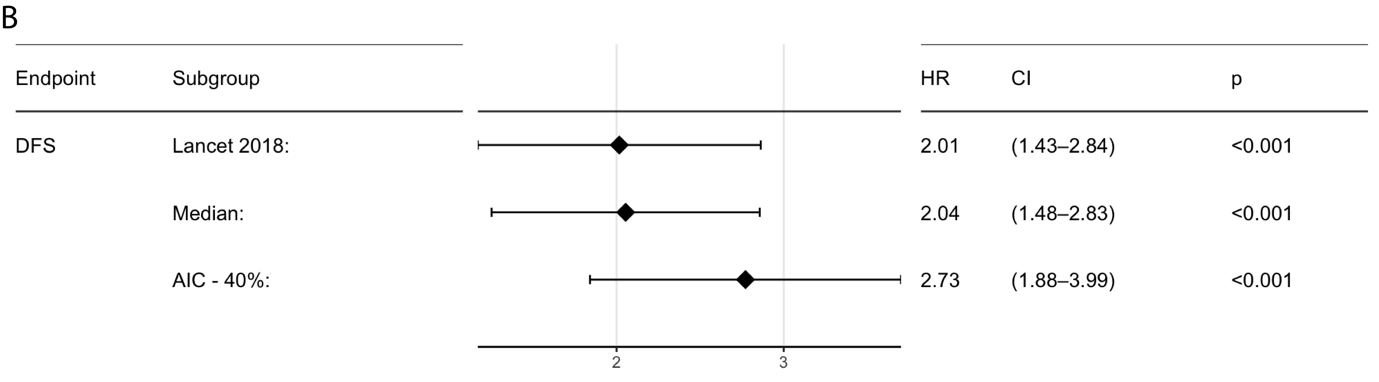
**

**Supplementary Figure 2– Analysis of the impact of TB (A) and Immunoscore (B) on patient outcome using different cut-offs.** In the main manuscript the international standards for Immunoscore and TB are used. Here a median cut-off and a cut-off based on lowest Akaike information criterion (AIC) are also used in univariate Cox regression analysis.

**
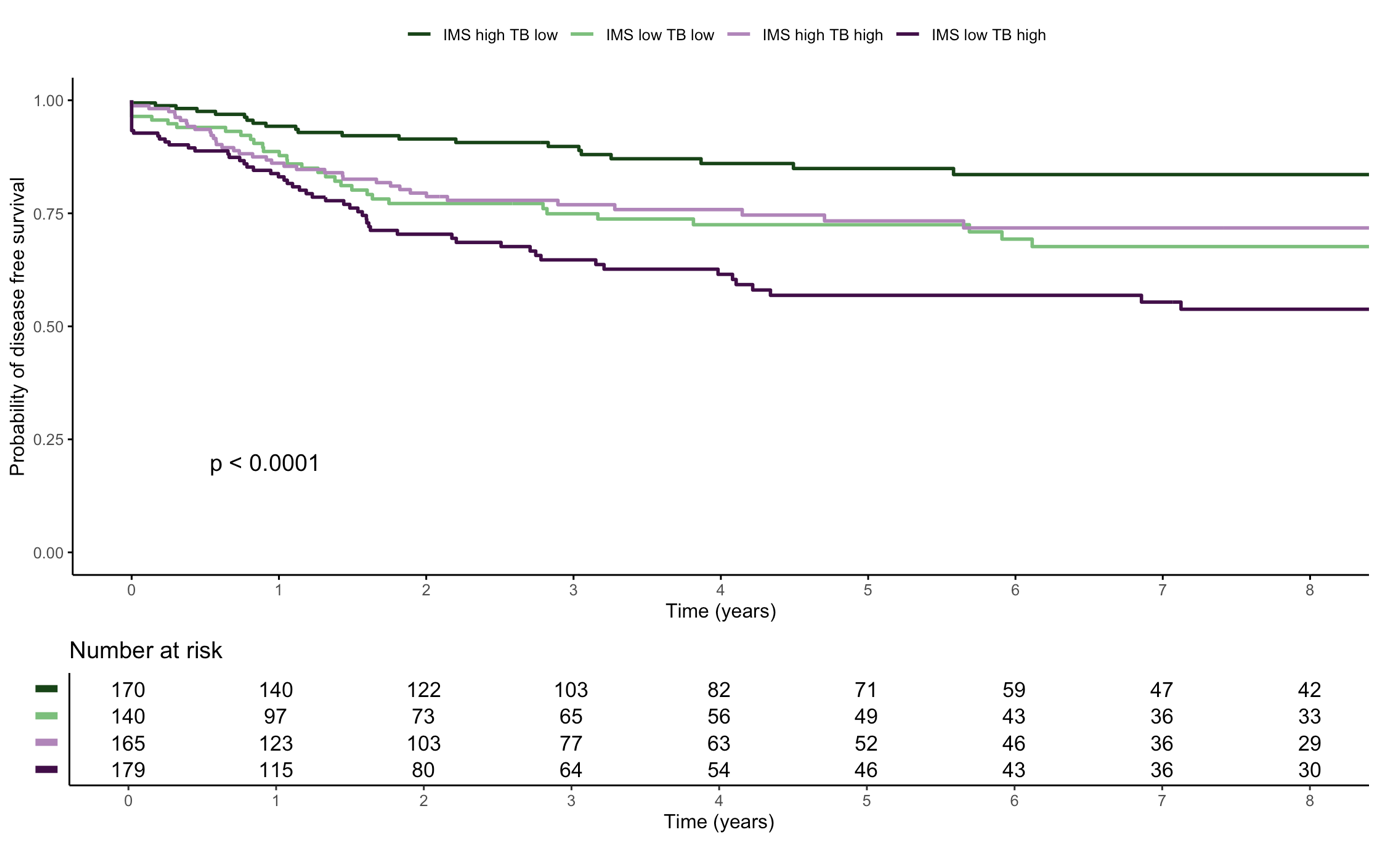
**

**Supplementary Figure 3 – Kaplan-Meier estimates of disease-free survival for patients with colon cancer grouped into four groups based on median of TB and Immunoscore scores.** The p-value obtained by the log rank test is included in the plot.

**
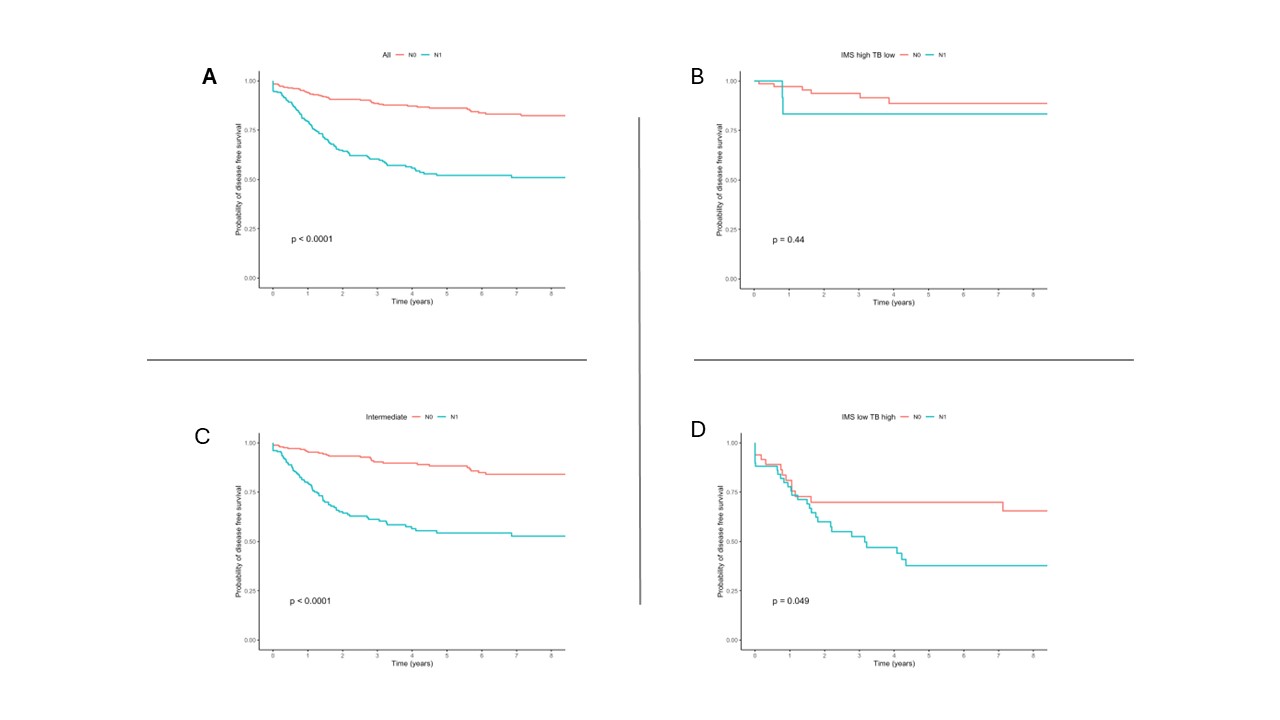
**

**Supplementary Figure 4- Kaplan-Meier estimates of disease-free survival for patients with colon cancer for the different categories to evaluate consistency for nodal stage. A. Overall cohort, B. Immunoscore high, TB low, C. Intermediate, D, Immunoscore low, TB high.** The p-value obtained by the log rank test is included in the plot.

**
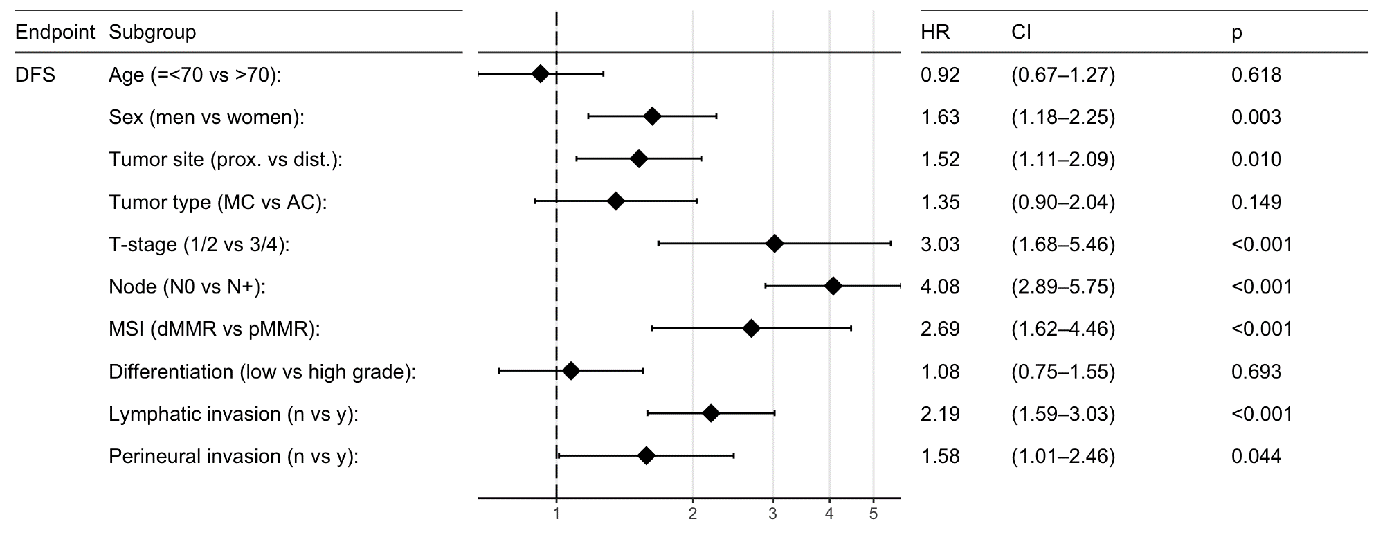
**

**Supplementary Table 1 - Forest plot based on results of univariable analysis of variables associated with DFS in overall cohort.**

**
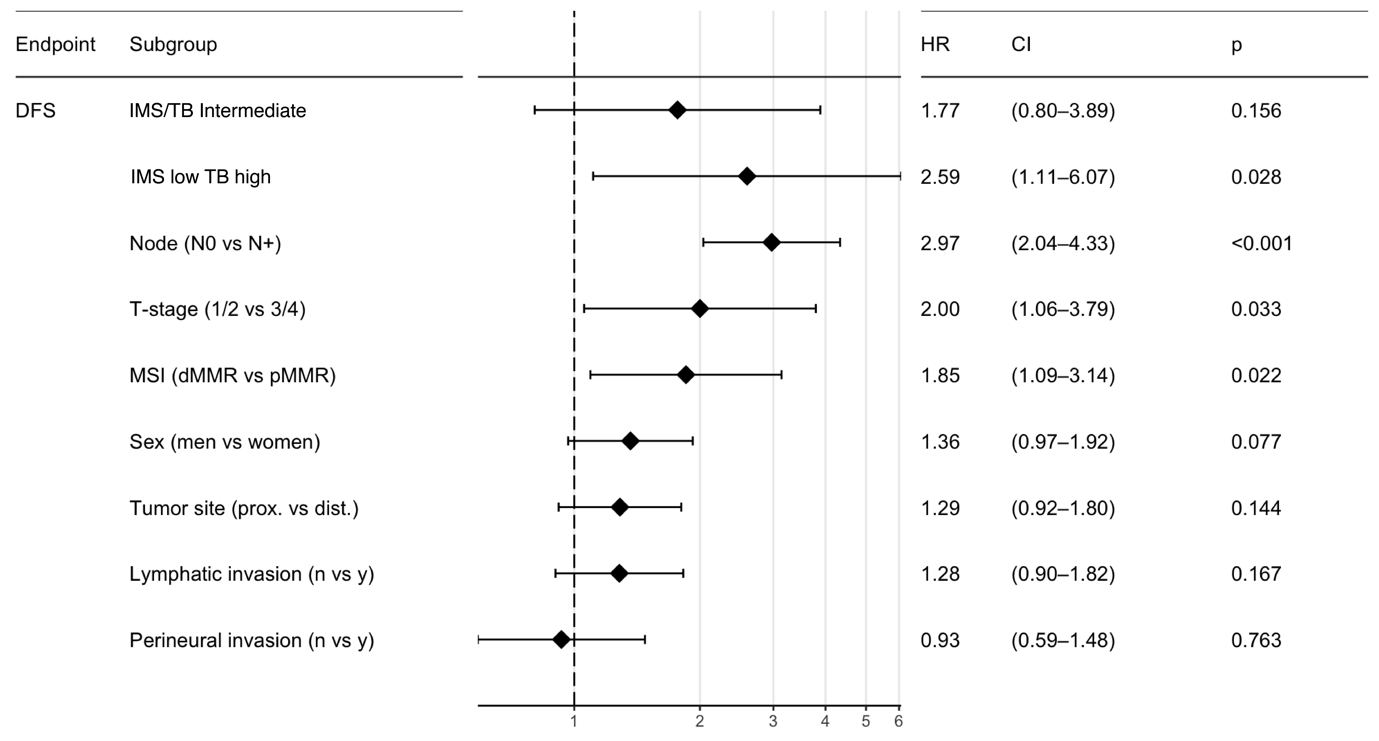
Supplementary Table 2 – Forest plot based on results of multivariate analysis of variables associated with DFS in overall cohort.** Variables significant in supplementary table 1 are included in this model.

**
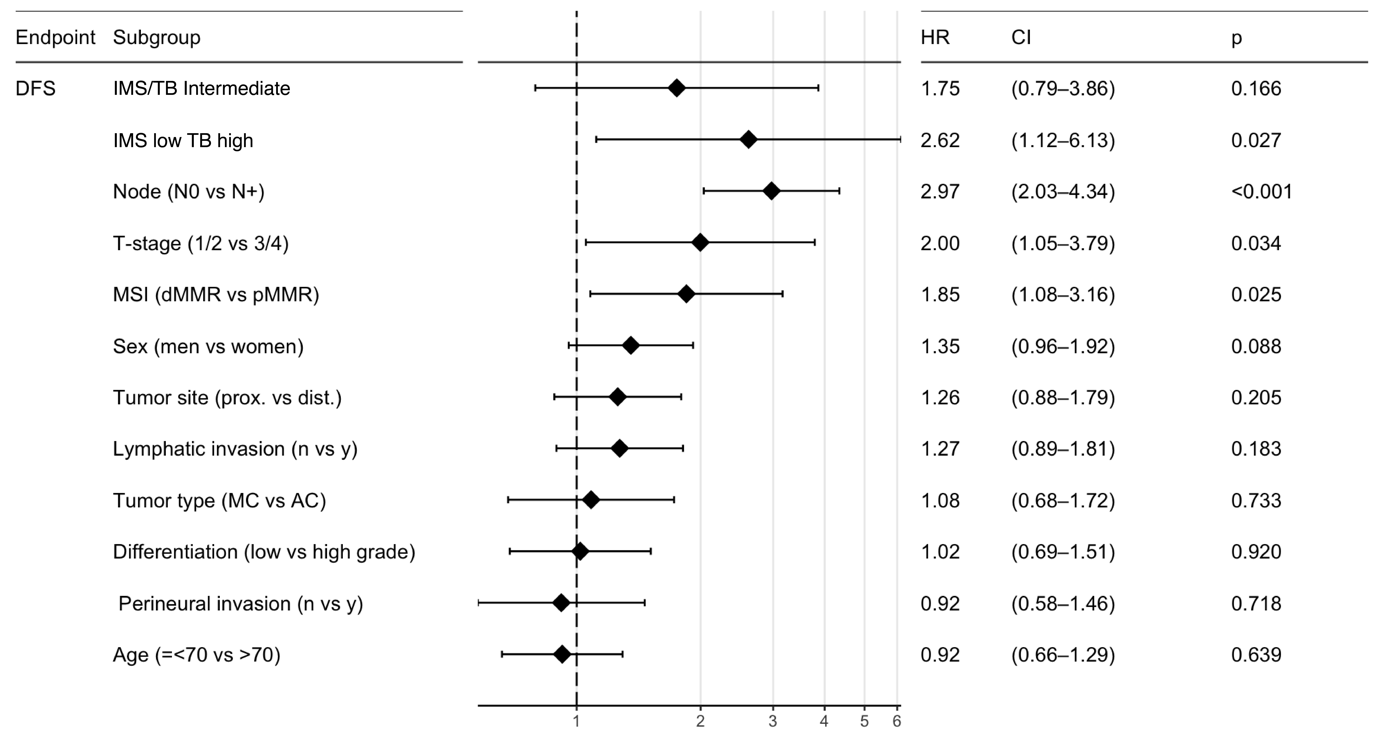
Supplementary Table 3 - Forest plots associated with figure 4A.** All variables were included in this multivariate Cox regression model.

**
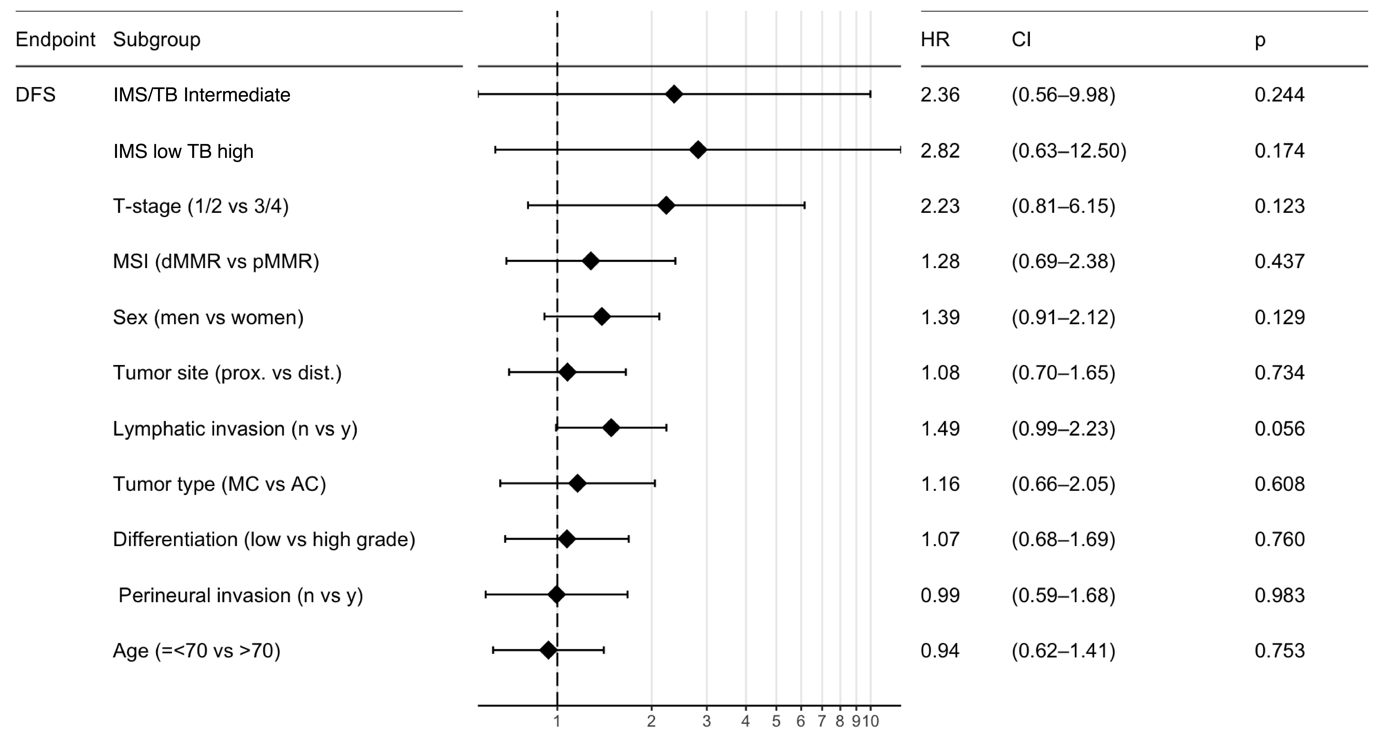
Supplementary Table 4 - Forest plots associated with figure 4B.** All variables were included in this multivariate Cox regression model.

**
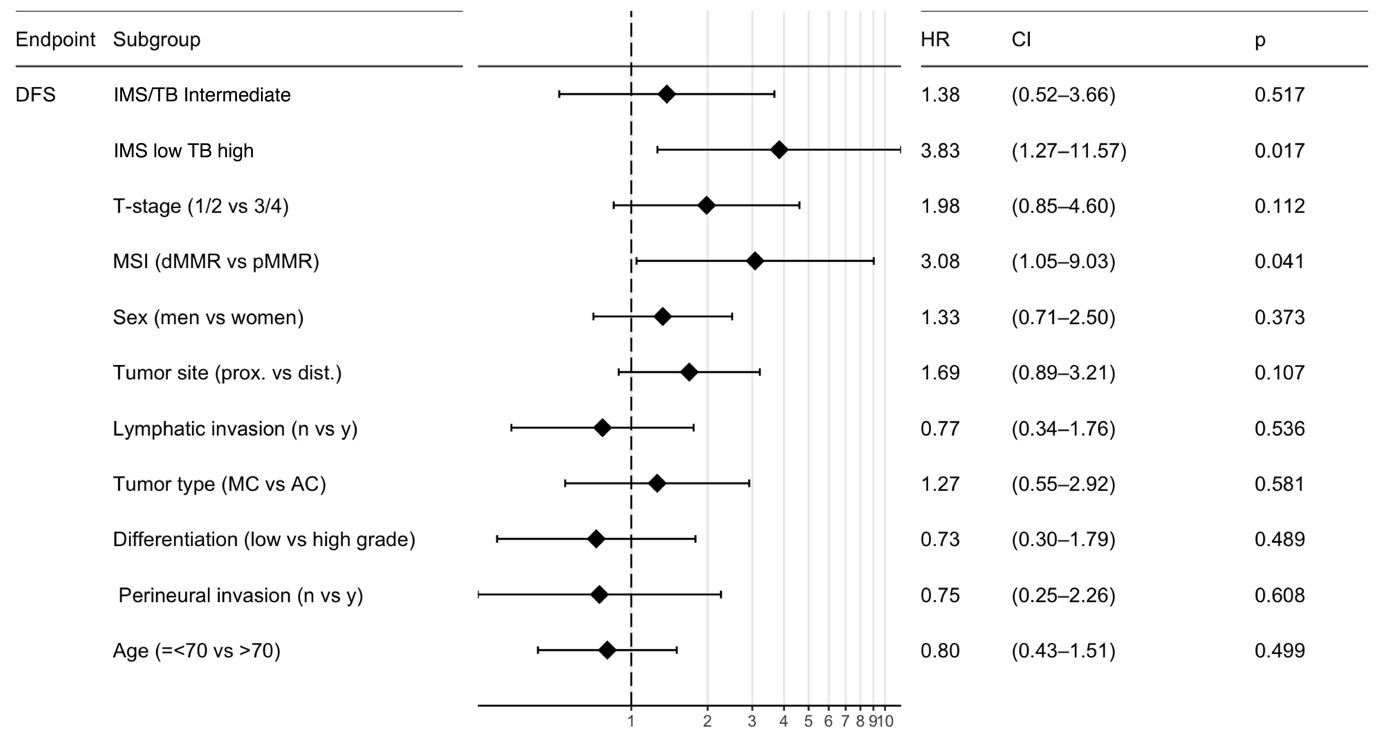
Supplementary Table 5 - Forest plots associated with figure 4C.** All variables were included in this multivariate Cox regression model.
